# Nutritional, antioxidative, and antimicrobial analysis of the Mediterranean hackberry (*Celtis australis* L.)

**DOI:** 10.1002/fsn3.375

**Published:** 2016-04-27

**Authors:** Ajda Ota, Ana Miklavčič Višnjevec, Rajko Vidrih, Željko Prgomet, Marijan Nečemer, Janez Hribar, Nina Gunde Cimerman, Sonja Smole Možina, Milena Bučar‐Miklavčič, Nataša Poklar Ulrih

**Affiliations:** ^1^ Biotechnical FacultyUniversity of LjubljanaJamnikarjeva 101LjubljanaSI‐1000Slovenia; ^2^ Science and Research Centre of KoperUniversity of PrimorskaZelena ulica 8IzolaSI‐6310Slovenia; ^3^ Agricultural Department in PorečUniversity of RijekaCarla Huguesa 6PorečCroatia; ^4^ Institute Jožef StefanJamova cesta 39LjubljanaSI‐1000Slovenia; ^5^ Centre of Excellence for Integrated Approaches in Chemistry and Biology of Proteins (CIPKeBiP)Jamova cesta 39LjubljanaSI‐1000Slovenia

**Keywords:** Antimicrobial activity, *Celtis australis*, nutritional analysis, phenols, triple‐quadrupole tandem mass spectrometry, ultrahigh‐pressure liquid chromatography

## Abstract

*Celtis australis* is a deciduous tree commonly known as Mediterranean hackberry or the European nettle tree. The fruit of hackberry are seldom used for nutritional purposes. The nutritional and physicochemical properties of ripe hackberry fruit from Istria (Marasi village near Vrsar, Croatia) were determined, including water, total fiber, protein, vitamin, mineral, and phenolic contents. This analysis demonstrates that the hackberry fruit is a valuable source of dietary fiber, protein, and vitamins, and of pigments such as lutein, *β*‐carotene, zeaxanthin, and tocopherols. The seasonal differences associated with the different growth stages for the element composition, total phenolic content, and phenolic profile were also determined for hackberry mesocarp and leaves. Water and ethanol extracts were prepared from mesocarp and leaves harvested at different growth stages and their phenolic profiles and antioxidant and antimicrobial activities were investigated. This study demonstrates that water and ethanol extracts of hackberry fruit and leaves collected at different growth stages contain epicatechin, gallic acid, vanillic acid, 3,4‐dihydroxybenzaldehyde, delphinidin‐3,5‐di‐O‐glucoside, cyanidin‐3,5‐di‐O‐glucoside, and pelargonidin‐3,5‐di‐O‐glucoside. They also show some antimicrobial and antifungal activities. Further studies are needed to identify and define the active ingredients of these hackberry leaf ethanol extracts.

## Introduction

*Celtis australis* L. is known as the Mediterranean hackberry, European nettle tree, or the lote tree, and it grows wild in mild Mediterranean regions (e.g., southern Europe, northern Africa) and in southeast Asia (Baytop 1994). It was initially placed into the *Ulmaceae* family, but was later re‐classified into the *Cannabaceae* family (Sytsma et al. 2002). The genus *Celtis* comprises about 70 species that are valued mainly for their wood (Bean 1981); (Chiej 1984); (Facciola 1990). In eastern North America, the hackberry *Celtis occidentalis* prevails, while the more humid soils in central North America are dominated by the shorter sugarberry tree, *Celtis laevigata* (Demır et al. 2002). Although the Mediterranean hackberry grows better in deep moist soils, it can often be found in poor soils among dry rock (Costa et al. 1998).

The fruit of the Mediterranean hackberry ripen in autumn as spherical drupes of 9–12 mm in diameter. These fruits are astringent in taste and contain a single seed that comprises approximately 38% of the fruit dry mass. The fruit has a high sugar content, which has been reported as up to 81.5% (Herrera 1987) and they are considered to be very effective medicinally due to their lenitive and stomachic properties (Chiej 1984; Chevallier 1996). Leaf and fruit preparations have preventive effects in the treatment of amenorrhea, colic, diarrhea, dysentery, peptic ulcers, and menstrual bleeding (Chevallier 1996; Chopra et al. 1986). The leaves of *Celtis australis* contain rare flavonoid c‐glycosides, such as acacetin 7‐O‐glucoside, isovitexin, and cytisoside (Spitaler et al. 2009; Zehrmann et al. 2010; Sommavilla et al. 2012).

A study into the nutritional and physical properties of hackberry fruit was carried out by Demır et al. (2002), who analyzed hackberry fruit for their crude oil, protein, crude fiber, and ash and mineral contents. The aim of this study was to initially determine the crude‐fiber, protein and fatty‐acid content in hackberry seeds, along with the sugar, vitamin (i.e., carotenes, tocopherols), and total phenol and mineral contents in the mesocarp and leaves of ripe hackberry fruit from Istria (Marasi village near Vrsar, Croatia). Total phenols were determined in water and ethanol extracts of these hackberry fruit. In addition, several phenolic compounds were identified using ultrahigh‐pressure liquid chromatography (UHPLC) linked to triple‐quadrupole tandem mass spectrometry (QqQ‐MS/MS). Also, as plant extracts are considered to be valuable sources of biologically active compounds that have antimicrobial activities, the antibacterial and antifungal activities against selected bacteria and fungi were determined for extracts from hackberry fruit mesocarp and leaves harvested at the different growth stages. To the best of our knowledge, this is the first study that includes determination of the nutritional and physicochemical properties along with the antibacterial and antifungal activities associated with the Mediterranean hackberry.

## Materials and Methods

### Materials

#### Chemicals and reagents

Folin‐Ciocalteu reagent, 2,2‐diphenyl‐1‐picrylhydrazyl (DPPH), RPMI medium, 2‐p‐iodophenyl‐3‐p‐nitrophenyl‐5‐phenyl tetrazolium chloride, gallic acid, lutein, *α*‐carotene, *β*‐carotene, and tocopherol were from Sigma, Germany. Mueller–Hinton broth and Mueller–Hinton Agar were from Oxoid, (Hampshire, UK), and zeaxanthin was from Applichem. Total Dietary Fibre kits (Merck, Darmstadt, Germany) were used for fiber determination. Standard methanol solutions of gallic acid (Sigma), vanillic acid (Fluka Chemika, Buchs, Switzerland), 3,4‐dihydroxybenzaldehyde (3,4‐dihydroxybenzaldehyde (DHA); Acros Organics), epicatechin (Sigma), delphinin‐3,5‐di‐*O*‐glucoside, cyanidin‐3,5‐di‐*O*‐glucoside, and pelargonidin‐3,5‐di‐*o*‐glucoside (Extrasythese, France) were used for determination of the phenolic compounds All other solvents, buffers, and reagents were analytically pure.

#### Plant material and extraction procedure

Unripe and ripe mature hackberry fruit and leaves were collected at different growth stages (i.e., end of June, end of October) in Istria (Marasi village near Vrsar, Croatia) and transported on the same day to the laboratory at the Biotechnical Faculty, University of Ljubljana, Slovenia. The fruit was deseeded and samples of mesocarp and seeds were lyophilized. Water and ethanol extracts from mesocarp and dry leaves of hackberry fruit were prepared by extraction of 5 g lyophilised powdered mesocarp and leaves in water and in 70% aqueous ethanol for 4 h. The extracts were centrifuged, dried by rotary evaporation, lyophilized, and kept at −20°C until use.

### Methods

#### Nutritional properties

Nutritional properties of the hackberry fruit mesocarp and seeds were determined according to the AOAC International reference methods (AOAC Official Method, 1997). Soluble solids were measured using a hand refractometer (Atago, Tokyo, Japan). The reducing sugars were determined using the Luff‐Schoorl method (Alexander et al. 1985). Total, soluble, and insoluble fibers were determined according to the modified enzymatic–gravimetric method of Prosky, using Total Dietary Fiber kit (Merck, Germany). The analysis was performed according to the manufacturer's instructions. The soluble and insoluble dietary fiber residues minus the ash and crude protein in the residues were taken as the respective dietary fiber fraction. The total dietary fiber was calculated as the sum of the soluble and insoluble dietary fiber.

#### Mineral element composition

The mineral element compositions of the ripe mesocarp and leaves were analyzed using energy dispersive X‐ray fluorescence spectrometry. From 0.5 g to 1.0 g of powdered samples were used to prepare pellets, using a pellet die and a hydraulic press. The disc radioisotope excitation sources of ^55^Fe (25 mCi) and ^109^Cd (20 mCi) (Eckert & Ziegler, Berlin, Germany) were used as the primary excitation sources. The emitted fluorescence radiation was measured using an energy dispersive X‐ray spectrometer composed of a Si(Li) detector (Canberra), a spectroscopy amplifier (Canberra M2024), an analog‐to‐digital converter (Canberra M8075), and PC‐based multichannel analyzer (S‐100, Canberra). The spectrometer was equipped with a vacuum chamber. The energy resolution of the spectrometer was 175 eV at 5.9 keV.

Analysis of the complex X‐ray spectra was performed using the AXIL spectral analysis program (Nečemer et al. 2008, 2011);. The evaluated uncertainty of this procedure included the statistical uncertainty of the measured intensities and the uncertainty of the mathematical fitting procedure. The overall uncertainties of the spectral measurements and the analyses were in most cases better than 1%. Quantification was then performed using the quantitative analysis of environmental samples software that was developed in our laboratory (Nečemer et al. 2008, 2011). The estimated uncertainty of the analysis was 5–10%. This relatively high total estimated uncertainty is mainly due to the contributions of the matrix correction and geometry calibration procedures, which included errors in the tabulated fundamental parameters, and also error contributions from the spectrum acquisition and analysis.

#### Lutein and *β*‐carotene contents

The lutein and *β*‐carotene contents of the ripe hackberry fruit mesocarp were determined according to the method described by (Sircelj and Batic 2007). Here, about 0.1 g lyophilized sample was mixed with 5 mL cold acetone. This mixture was homogenized using an Ultra‐Turrax (Janke & Kunkel GmBH & Co., Staufen, Germany) at 20,000 rpm for 20 sec, and centrifuged at 2300 g for 5 min. The supernatant was then filtered through a 0.45 *μ*m filter, and the tocopherol content was determined using high‐performance liquid chromatography (HPLC; Spectra‐Physics, Santa Clara, CA, USA), with a P4000 pump (SpectraSYSTEM), an AS1000 autosampler (SpectraSYSTEM) and a UV‐Vis detector. The samples were injected into a precolumn (Spherisorb ODS2 5U; 5 *μ*m, 50 × 4.6 mm), from where they passed into the main column (Spherisorb ODS2 5U; 5 *μ*m, 250 × 4.6 mm). The injection volume was 20 *μ*L. The mobile phase comprised acetonitrile/ H_2_O/ methanol (A; 100:10:5, v/v/v) and acetone/ ethylacetate (B; 2:1, v/v) with a gradient of B in A of 10–75% in 18 min, 75–70% in 7 min, and 70–100% in 5 min. The flow rate was 1 mL/min. The column temperature was 5°C (Mistral tip 880, Spark Holland) and the autosampler temperature was 4°C. The detection wavelength was 440 nm, and the time of the analyses was 30 min. Lutein and *β*‐carotene were identified by comparison of their retention times with those of authentic standards (Sigma). Quantification was based on the external standard method.

#### Tocopherols contents

The extraction procedure for the tocopherols was the same as that described for lutein and *β*‐carotene, and the tocopherols contents were determined according to the method described by Wildi and Lutz (1996), using fluorescence detection after chromatography. The same HPLC system was used as that for the determination of lutein and *β*‐carotene (i.e., Spectra‐Physics HPLC system), and the parameters used were the same, except that the mobile phase was methanol, the column temperature was 20°C, and the detection used an excitation wavelength of 295 nm and an emission wavelength of 325 nm. The tocopherols were identified by comparisons of their retention times with those of authentic standards (Sigma). The quantification was based on the external standard method.

#### Fatty‐acid composition

The fatty acids were determined as their methyl esters after transesterification, according to AOAC Official Method, (1997), using gas chromatography (HP 5890, series II; Hewlett Packard Corp., Palo Alto) equipped with a fused‐silica capillary column (SP2380; Supelco; 30 m × 0.25 mm; film thickness, 0.20 μm). The stationary phase was poly(biscyanopropyl/ cyanopropylphenyl siloxane) (9:1, v/v). The carrier gas was helium, at a flow rate of 1 cm^3^/min. The internal standard was heptadecanoic acid. The column temperature was set for 150–210°C at 5°C/min. The injector and flame‐ionization detector temperatures were set at 220°C and 250°C, respectively. The injection volume was 1 *μ*L. Identification was achieved by comparison of the retention times for the fatty acid methyl esters (FAMEs) of the investigated oil samples with the retention times of FAME standards (FAME Mix rapeseed oil). The data are given as weight percentages of the total fatty acids. The analyses were carried out in duplicate. The standard errors of determination were between 0.01% and 0.25%.

#### Total phenolics

The total content of phenolic compounds in the mesocarp and leaf extracts of *C. australis* were determined spectrophotometrically (Waterman and Mole 1994). An appropriately diluted extract or gallic acid (as the calibration standard) was mixed with freshly prepared Folin‐Ciocalteau reagent, sodium carbonate solution (20%, w/v), and Milli‐Q water. After 40 min, the total phenolic compounds in the extracts of the mesocarp and leaves were determined at 765 nm. The total amounts of phenolic compounds are expressed per gram gallic acid. The analyses were conducted in triplicate, with the data given as means ± SEM.

#### Identification of phenolics by *UHPLC*–*QqQ*‐*MS/MS*

For the identification of the phenolic compounds, the freeze dried extracts were reconstituted with ultrapure water (Milli‐Q) and ethanol. The phenolic compounds were identified and determined, using UHPLC interfaced to QqQ‐MS/MS, on an HPLC system (Agilent 1260) equipped with a degasser (model G4225A), a binary gradient pump (model G1312A,) a thermoautosampler (model G1329B), a column oven (model G1316A), a diode array detection system (model G4212B), and a Poroshell 120 column (EC‐C18; 2.7 *μ*m; 3.0 × 50 mm). Elution was with a gradient of water/ formic acid (99.5:0.5; v/v) (A) and acetonitryl /methanol (50:50; v/v) (B) as follows: 0 min, 96% A/ 4% B; 3.96 min, 50% A/ 50% B; 4.45 min, 40% A/ 60% B; 5.94 min, 0% A/ 100% B, which was held until completion at 7.13 min. In addition, a further HPLC column was used (Ascentis; 2.7 *μ*m; 15 cm × 4.6 mm) with a gradient of water/ formic acid (99.5:0.5; v/v) (A) and acetonitryl/ methanol (50:50; v/v) (B) that was adjusted properly according to the retention time. The flow was 0.2 mL/min, and the sample injection volume was 1 *μ*L. The HPLC system was connected in series with a triple quadrupole mass spectrometer (6420; Agilent Technology, Santa Clara, CA, USA). The standard phenolic compounds were dissolved in methanol and infused, to optimize the acquisition parameters. The MS and MS/MS spectra of the compounds under investigation were acquired using the following optimized conditions: fragmentor voltage, 80–220 V; collision energy, 15–45 eV; capillary voltage, 4.0 kV; cell acceleration voltage, 7 V; sheath gas temperature, 400°C; cone gas flow, 10 L/min; desolvation gas flow, 12 L/min; and collision gas, nitrogen. For multiple reactions, monitoring (MRM) of the individual compounds, the positive ion (anthocyanins, system I) and negative ion (nonanthocyanin phenolic compounds, system II) modes were selected for the first quadrupole, and the collision energies were adjusted to optimize the signal for the most abundant product ions. The limit of detection (LOD) of the method was 0.04 *μ*g/100 g sample for epicatechin, 0.002 *μ*g/100 g sample for gallic acid, 0.001 *μ*g/100 g sample for vanillic acid, 0.04 *μ*g/100 g sample for 3,4‐DHA, and 2 *μ*g/100 g sample for delphinidin‐3,5‐di‐O‐glucoside, cyanidin‐3,5‐di‐O‐glucoside, and pelargonidin‐3,5‐di‐O‐glucoside. The determined levels of delphinidin‐3,5‐di‐O‐glucoside, cyanidin‐3,5‐di‐O‐glucoside and pelargonidin‐3,5‐di‐O‐glucoside were calculated on the basis of calibration curves constructed using their respective chlorides.

#### Antioxidant activity

The antioxidant activities of the ethanol and water extracts of the hackberry mesocarp and leaves from different growth stages were determined spectrophotometrically, using the DPPH reagent, 2,2‐diphenyl‐1‐picryl‐hydrazyl (Brand‐Williams et al. 1995). The analyses were performed in triplicate for each extract.

#### Antibacterial activity

The antibacterial activities of the ethanol and water extracts of the hackberry mesocarp and leaves from different growth stages were tested against a panel of pathogenic microorganisms, including Gram‐positive *Staphylococcus aureus* ATCC 25923 and *Listeria monocytogenes* ŽMJ58a, and Gram‐negative *Escherichia coli* O157:H7 ŽMJ370 and *Pseudomonas aeruginosa* ŽMJ87 (all clinical isolates). The cultivation/ assay media were Mueller–Hinton broth or Mueller–Hinton agar (Oxoid, Hampshire, UK). The minimum inhibitory concentrations (MICs) for the extracts were determined using the broth microdilution method, as previously described (Klančnik et al. 2010). Briefly, 50 *μ*L of each bacterial suspension in suitable growth medium was added to the wells of a sterile 96‐well microtiter plate that already contained 50 *μ*L of twofold serially diluted plant extracts in the relevant growth medium. The final volume in each well was 100 *μ*L. Control wells were prepared with culture medium, bacterial suspension only, plant extracts only, and ethanol at the concentration corresponding to the highest concentration present. The MICs were the lowest concentrations where no viability was observed after 24 h on the basis of the metabolic activity. To indicate respiratory activity, the presence of color was determined after addition of 10 *μ*L/well 2 mg/mL 2‐p‐iodophenyl‐3‐p‐nitrophenyl‐5‐phenyl tetrazolium chloride (Sigma) dissolved in water, and an incubation under the appropriate cultivation conditions for 30 min in the dark (Klančnik et al. 2010). All of the measurements of the MICs were carried out in duplicate.

#### Antifungal activity

The antifungal activities of only the ethanol extracts of the hackberry mesocarp and leaves were tested against eight ubiquitous opportunistic pathogenic fungi: *Saprochaaete clavata* EX 5631, *Candida albicans* EX 9382, *Candida papapsilosis* EX 9370, *Aureobasidium pullulans* EX 3105, *Aspergillus fumigatus* EX 8280, *Fusarium dimerum* EX 9214, *Exophiala dermatitidis* EX 5586, and *Rhodotorula mucilaginosa* EX 9762, as the most frequent fungal colonizers of dishwashers (Zalar et al. 2011; (Gümral et al. 2015) and washing machines (Babič et al. 2015) around the world. All of the fungal cultures are deposited in the Ex Culture Collection of Extremophilic Fungi, as part of the Infrastructural Centre Mycosmo (MRICUL) at the Department of Biology, Biotechnical Faculty, University of Ljubljana, Slovenia. The tests were carried out according to the M27‐A3 and M38‐A2 reference methods for broth dilution antifungal susceptibility testing of yeast (CLSI 2008a) and filamentous fungi (CLSI 2008b), respectively. For the broth microdilution test, 50 *μ*L of each inoculum suspension with final cell counts of approximately 10^4^ cells/mL in RPMI medium was added to the wells of a sterile 96‐well microtiter plate that already contained 50 *μ*L twofold serially diluted plant extracts in the relevant growth medium. The final volume in each well was 100 *μ*L. The MICs were determined after 48 h incubation at 30°C. The MICs of the antifungal agents are defined as the lowest concentrations that inhibited visible fungal growth. All of the measurements of the MICs were carried out in duplicate.

## Results and Discussion

### Nutritional properties of hackberry fruit

The nutritional properties of hackberry fruit are given in Tables 1 and 2. The data for water, soluble solids, and reducing sugars in the air‐driedmesocarp, and for total fat in the air‐dried seeds are given in Table 1. The mesocarp of the ripe hackberries contained 30.0% water, while that of the seeds was 18.1% in the air‐dried samples (Table 1). The fruit contained 3.4 g/100 g protein and 10.2% total dietary fiber, 8.2% insoluble fiber, and 2.0% soluble fiber. According to Demır et al. (2002), the mesocarp contains 90.2% dry matter, which is higher than that determined in the present study. The difference in the water contents is probably due to loss of water prior to harvest.

**Table 1:** Water, soluble solids, and reducing sugars in air‐dried mesocarp and total fat in air‐dried seeds

Analysis	Mesocarp	Seeds
Water (%)	30.0 ± 1.2	18.1 ± 0.98
Soluble solids (%Brix)	53.4 ± 0.1	a
Reducing sugars (g/100 g fw)	50.9 ± 0.19	a
Total fat (g/ 100 g dw)	a	8.1 ± 1.0

Data are means ± SD.

fw, fresh weight, dw, dry weight.

a not measured.

**Table 2:** Mineral content in hackberry fruit mesocarp and leaves

Element	Mesocarp (mg/100 g dw)	Leaves (mg/100 g dw)	
	October	June	October
Si	ND	1280 ± 135	2570 ± 162
P	143 ± 2	141 ± 1	131 ± 18
S	20.8 ± 3.2	102 ± 17	84 ± 8
Cl	71 ± 4.9	240 ± 24	343 ± 21
K	1060 ± 64	834 ± 75	975 ± 59
Ca	269 ± 16	5970 ± 537	5590 ± 339
Mn	1.2 ± 0.01	6.0 ± 0.54	11.8 ± 3.0
Fe	5.1 ± 0.4	11.4 ± 0.6	17.8 ± 2.6
Ni	0.25 ± 0.09	0.24 ± 0.09	1.72 ± 0.75
Cu	0.45 ± 0.06	1.09 ± 0.09	2.01 ± 0.47
Zn	0.35 ± 0.07	0.79 ± 0.08	1.03 ± 0.01
Se	0.09 ± 0.01	0.08 ± 0.01	0.34 ± 0.01
Pb	0.07 ± 0.03	0.16 ± 0.03	0.70 ± 0.25
Br	0.69 ± 0.04	0.96 ± 0.05	2.01 ± 0.18
Rb	1.04 ± 0.09	0.32 ± 0.04	0.56 ± 0.18
Sr	0.50 ± 0.03	6.00 ± 0.26	5.92 ± 0.30
Mo	0.04 ± 0.01	0.04 ± 0.01	0.17 ± 0.01

Data are means (*n* = 2) ± SD.

ND, not detected.

Table 2 gives the mineral contents in the mesocarp of ripe hackberry and the leaves collected during the different growth stages (i.e., end of June, end of October), expressed per 100 g dry weight. Among the minerals, K was at the highest concentration in mesocarp, followed by Ca and P. In the leaves collected at the same time, the Ca content (5.59 g/100 g) reached more than fivefold that of K. The major difference between the leaves collected at the different growth stages at the end of June and the end of October was seen for Si, the content of which increased twofold over this 4‐month period.

For the microelements in mesocarp, Fe was the major microelement at 5.05 mg/100 g, followed by Mn (1.20 mg/100 g) and Rb (1.04 mg/100 g), and slightly lower concentrations for Br (0.69 mg/100 g), Cu (0.45 mg/100 g) and Zn (0.35 mg/100 g) (Table 2). Only one literature source was found regarding mineral contents (Demır et al. 2002). These samples of Demır et al.( 2002) contained threefold more K, slightly more P, and one‐sixteenth of the Ca levels. With regard to the microelements also determined by Demır et al. (2002); our samples contained similar amounts of Zn, slightly more Pb, Cu, and Ni, and twice as much Fe and Mn. The mineral contents of fruit depend on genetic factors and pedoclimatic conditions. The hackberry trees from which the fruit was harvested in the present study were growing wild, with no agricultural practices applied. We hypothesize that the main reason for the differences observed between this study and the study of Demır et al. (2002) lies in the different pedoclimatic conditions, although no mineral analyses of the soils were carried out in either case. The reason for the much higher content of Na in the fruit in this study might be the proximity of the sea. In comparison to mesocarp, leaves contained more than 20‐fold more Ca (5.97 g/100 g), with this as the predominant element, followed by Si, which was not detected in the mesocarp, and K. The element compositions of leaves changed depending on the growth stage. The major difference that was observed was the increase in Si levels from 1.28 g/100 g to 2.57 g/100 g from the end of June to the end of October.

Regarding the pigments and tocopherols in the mesocarp of the ripe hackberry fruit, among the carotenoids, *β*‐carotene was the most abundant (5.58 *μ*g/g), followed by lutein (1.41 *μ*g/g) and zeaxanthin (0.74 *μ*g/g). The mesocarp of hackberry fruit also contained *α*‐, *γ*‐ and *δ*‐tocopherols. Here, *α*‐tocopherol was the most abundant among the tocopherols, at 150.13 *μ*g/g dry matter (dm), while *γ*‐tocopherol and *δ*‐tocopherol were at 4.9 *μ*g/g dm and 2.8 *μ*g/g dm, respectively. For the pigments, hackberry contained 3.7 *μ*g/g dm chlorophyll a. In the literature, there appears to be the single report for carotenoids of 7.5 *μ*g/g in hackberry fruit (Boudraa et al. 2010), which is higher in comparison to values for *β*‐carotene determined in the present study.

Regarding the fatty acid composition, the seeds of these hackberry fruit contained mainly linoleic acid, at 5.109 g/100 g seeds, which represented 76.25% of all of the fatty acids. The remaining main fatty acids were oleic acid (0.951 g/100 g seeds; 14.18%), palmitic acid (0.452 g/100 g seeds; 6.72%), and stearic acid (0.188 g/100 g seeds; 2.81%). No data for the fatty acid composition of hackberry seeds were found in the literature.

The visual and textural appearance of the hackberry mesocarp resembles that of dried dates and dried figs. When the chemical composition of hackberry fruit determined here was compared to a previous study on fig, these hackberries contained similar amounts of total fibre and protein, but fivefold greater amont of *β*‐carotene (Saxholt et al. 2008);. Hackberry also contained a similar mineral chemical composition to figs, in particular with respect to K, Ca, and P (Saxholt et al. 2008).

### Total phenolic compounds

When the water extracts were examined, for the ripe mesocarp they contained 0.27 g/100 g total phenols (as gallic acid equivalents), and for unripe mesocarp they contained 0.05 g/100 g total phenols. The total phenols determined in the ethanol extract of ripe mesocarp gave 0.24 g/100 g total phenols, while that for the unripe mesocarp contained 0.10 g/100 g total phenols. Compared to dates (*Phoenix dacttyfera* L.), the present analyses of ripe hackberry mesocarp showed comparable total phenols (Saafi et al. 2009).

Contrary to literature data (Sommavilla et al. 2012), seasonal differences were observed for total phenolics content between leaves harvested at the different growth stages at the end of June and at the end of October. Water extracts contained higher amounts of total phenols in the ripe mesocarp (i.e., end of October; 0.27 g/100 g), while this was lower in the unripe mesocarp (i.e., end of June; 0.05 g/100 g). The water extracts of leaves collected at the end of October also contained more than twofold higher concentrations of phenols (0.17 g/100 g) than leaves harvested at the end of June (0.08 g/100 g). The total phenols content in the water extract of leaves was comparable to that reported for *Ficus carica* leaves (Konyalιoğlu et al. 2005. The total amount of phenols determined in the ethanol extract of leaves collected at the end of June was 0.07 g/100 g total phenols, while their concentration increased 10‐fold (to 0.89 g/100 g) by the end of October.

In addition to their function as antioxidants, the phenolic compounds act as screening agents against effects of UV‐B radiation and water deficit, which potentially explains the increased phenolics contents in mesocarp and leaves harvested at the end of October (Grace 2005; Petridis et al. 2012).

**Figure 1:**
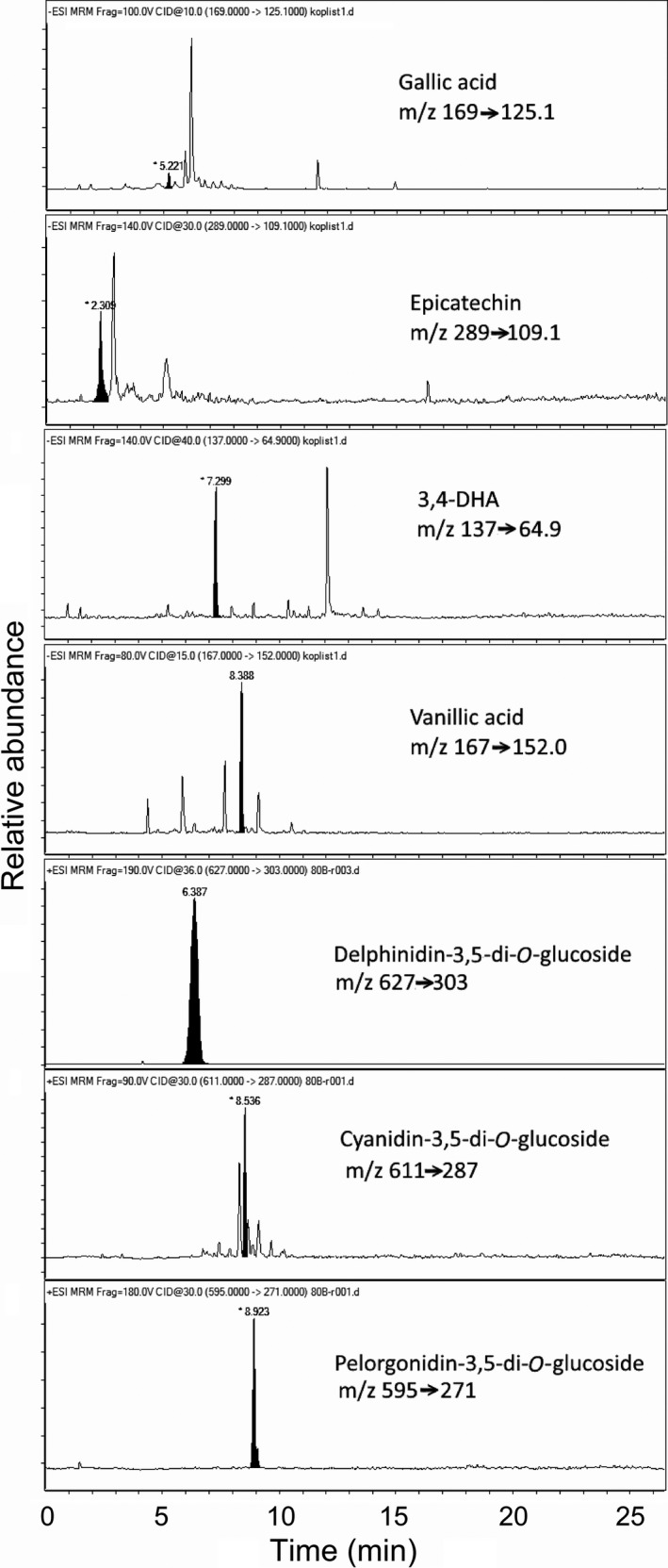
Representative chromatograms (as indicated) from combined ultrahigh‐pressure liquid chromatography and triple‐quadrupole tandem mass spectrometry of phenols in *Celtis australis*.

The individual phenolic compounds identified in the water and ethanol extracts obtained from mesocarp and leaves (Fig. 1) harvested at different growth stages are presented in Table 3. The levels of these phenolic compounds were generally lower in the water extracts compared to the ethanol extracts. The predominant phenolic compound in the present study was in the ethanol extracts of mesocarp of both unripe and ripe hackberry fruit, which was cyanidin‐3,5‐di‐O‐glucoside; this was followed by pelargonidin‐3,5‐di‐O‐glucoside and 3,5 DHA, with a ripe mesocarp containing at least 10‐fold more than the unripe mesocarp. Pelargonidin‐3,5‐di‐O‐glucoside, cyanidin‐3,5‐di‐O‐glucoside, 3,5 DHA, vanillic acid, and epicatechin were detected in the ethanol extract of leaves harvested in June. Over the next 4 months to the October sampling, the levels of epicatechin increased 100‐fold, while those of cyanidin‐3,5‐di‐O‐glucoside and vanillic acid almost doubled. However, more data need to be collected to confirm these seasonal variations in the phenolic compounds determined here. Previously acacetin 7‐O‐glucoside, isovitexin, and cytisoside were isolated from leaves of hackberry (Spitaler et al. 2009) although there are no data available for seasonal variations of pelargonidin‐3,5‐di‐O‐glucoside, cyanidin‐3,5‐di‐O‐glucoside, 3,5 DHA, vanillic acid, and epicatechin. The overall increases in the phenols during this 4‐month period will contribute to the protective effects of certain phenolic compounds against damage from UV‐B radiation and water deficit (Grace 2005; Petridis et al. 2012). Nevertheless, it should be noted that we have here defined only a few of the phenolic compounds that are present in the Mediterranean hackberry. Therefore, more phenolic compounds need to be identified and their levels determined.

**Table 3:** Total and individual phenolic compounds in the water and ethanol extracts from mesocarp and leaves harvested at the different growth stages

Extract	Compound	Mesocarp (mg/100 g fw)		Leaves (mg/100 g fw)	
		June	October	June	October
Water	Epicatechin	<LOD	<LOD	<LOD	<LOD
	Gallic acid	<LOD	0.114 ± 0.020	0.013 ± 0.003	<LOD
	Vanillic acid	<LOD	<LOD	<LOD	<LOD
	3,5‐Dihydroxybenzaldehyde	<LOD	<LOD	<LOD	<LOD
	Delphinidin‐3,5‐di‐O‐glucoside	<LOD	0.016 ± 0.001	<LOD	0.004 ± 0.000
	Cyanidin‐3,5‐di‐O‐glucoside	<LOD	<LOD	<LOD	0.003 ± 0.001
	Pelargonidin‐3,5‐di‐O‐glucoside	<LOD	0.018 ± 0.001	<LOD	0.007 ± 0.001
	Total phenolics	53.3 ± 0.4	274.4 ± 9.2	76.1 ± 0.9	168.3 ± 1.4
Ethanol	Epicatechin	<LOD	<LOD	0.133 ± 0.014	13.522 ± 0.655
	Gallic acid	<LOD	<LOD	<LOD	<LOD
	Vanillic acid	<LOD	<LOD	0.463 ± <LOD	0.784 ± 0.008
	3,5‐Dihydroxybenzaldehyde	0.373 ± 0.023	3.480 ± 0.030	1.183 ± 0. 024	1.011 ± 0.062
	Delphinidin‐3,5‐di‐O‐glucoside	<LOD	<LOD	<LOD	<LOD
	Cyanidin‐3,5‐di‐O‐glucoside	0.004 ± 0.001	0.051 ± 0.001	0.005 ± 0.001	0.011 ± 0.0010
	Pelargonidin‐3,5‐di‐O‐glucoside	0.002 ± 0.001	0.033 ± 0.001	0.007 ± 0.001	0.007 ± 0.001
	Total phenolics	101.6 ± 1.4	239.1 ± 3.2	74.1 ± 1.9	894.2 ± 21.2

Data are means ± SD.

fw, fresh weight; LOD, limit of detection.

### Antioxidative potential

**Figure 2:**
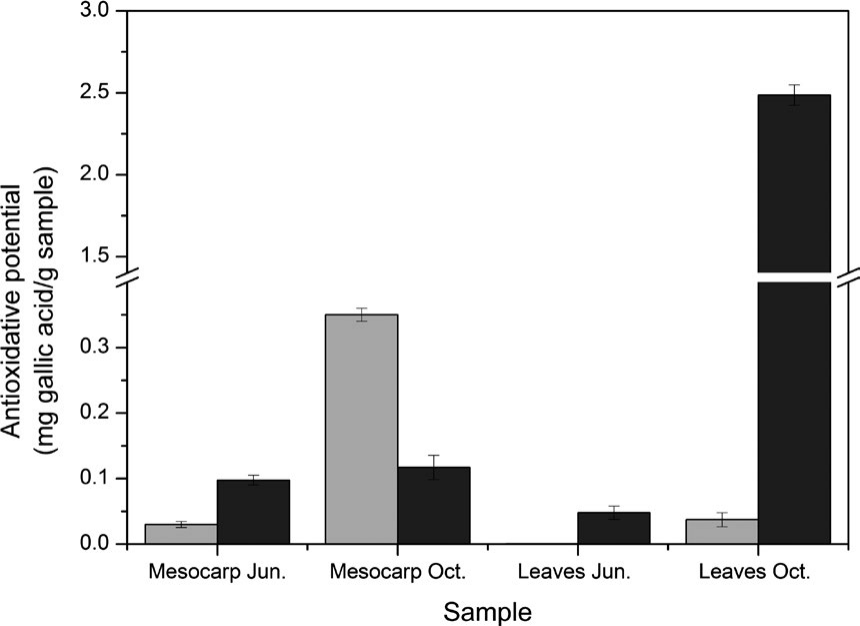
Influence of water (light gray) and 70% ethanol (dark gray) as the extraction medium on the antioxidative potential of hackberry mesocarp and leaves extracts collected at different growth stages.

As seen from Figure 2, the highest antioxidative potential (given as gallic acid equivalents) of mesocarp was in the water extract, followed by the ethanol extract, as also observed by Zehrmann et al. (2010). For the leaves, the ethanol extract harvested at the end of October contained 50‐fold the level of antioxidants than the leaves harvested at the end of June. This is consistent with the significantly increased levels of epicatechin (100‐fold), which shows a positive and high correlation with antioxidant capacity (Othman et al. 2010), and the doubled levels of cyanidin‐3,5‐di‐O‐glucoside and vanillic acid found.

### Antibacterial activity

Antibacterial activity tests were performed with the water and ethanol extracts of hackberry mesocarp and leaves collected at the different growth stages. The antimicrobial activities were measured against two Gram‐positive bacteria, *Staphylococcus aureus* and *Listeria monocytogenes*, and two Gram‐negative bacteria, *Pseudomonas aeruginosa* and *Escherichia coli* O157:H7. These data are given in Table 4.

**Table 4:** Antibacterial activities of water and ethanol extracts of hackeberry mesocarp and leaves collected at the different growth stages

Bacterium	Minimum inhibitory concentrations (mg/mL)							
	Water extract				Ethanol extract			
	Mesocarp		Leaves		Mesocarp		Leaves	
	June	October	June	October	June	October	June	October
*Escherichia coli*	>10	5	5	2.5	>10	5	2.5	5
*Listeria monocytogenes*	>10	>10	>10	10	>10	5	>10	5
*Pseudomonas aeruginosa*	>10	>10	>10	>10	>10	2.5	>10	>10
*Staphylococcus aureus*	>10	5	>10	5	>10	1.25	>10	1.25

The extracts prepared from the hackberry mesocarp and leaves harvested at the end of October showed low antibacterial activities, while those harvested earlier (i.e., in June) showed weaker, or in the case of the hackberry mesocarp, no antibacterial activity against these bacterial species. As previously described bySowkat et al. (2012), the water extract from leaves had a higher antibacterial activity against *S. aureus*, compared to *P. aeruginosa*. This was confirmed also in the present study with leaves collected in October; in addition, comparable activities were also found for the fruit mesocarp (Table 4). Badoni et al. (2010) also studied the antimicrobial activities of crude extracts from hackberry fruit and reported antibacterial activities against *S. aureus, B. subtilis, P. aeruginosa*, and *E. coli*. Although different extraction solvents were used and lower MICs were determined, the growth of *S. aureus* was again inhibited at lower concentrations than for *P. aeruginosa*. Indeed, the different methods of extraction and solvents used will be one of the reasons for the differences in these MICs. These data are comparable to the antibacterial activities of *Ficus carica* leaves, where weak antibacterial activity was shown against *E. coli* and *S. aureus* (*Jeong* et al. 2009).

### Antifungal activity

The most frequent fungal colonizers of both dishwashers and washing machines were selected to determine the antifungal activities of the ethanol extracts of mesocarp and leaves. These were *Saprochaaete clavata* EX 5631, *Candida albicans* EX 9382, *Candida papapsilosis* EX 9370, *Aureobasidium pullulans* EX 3105, *Aspergillus fumigatus* EX 8280, *Fusarium dimerum* EX 9214, *Exophiala dermatitidis* EX 5586, and *Rhodotorula mucilaginosa* EX 9762.

While the mesocarp ethanol extract showed no antifungal activities, extracts prepared from leaves harvested at the end of October inhibited the growth of *C. albicans*,* C. parapsilosis* (MICs, 0.156 mg/mL), and *R. mucilaginosa* (MIC, 0.313 mg/mL), although the growth of other tested fungi was not inhibited. These data can be explained by the significantly higher (i.e., 10‐fold) amounts of phenols in the ethanol extract of leaves. This is of particular importance as over the last 30 years the fungi in indoor environments have increasingly been recognized as a potential health problem, which is linked to the growingly immunocompromised population (de Vos et al. 2009; (Neofytos et al. 2007). Conditions inside household appliances were originally considered to be prohibitive for fungal growth; however, novel energy‐saving trends have led to the use of these appliances at lower temperatures and with biodegradable detergents. These conditions have thus become selective for thermo tolerant, oxidative stress resistant, and generally stress‐tolerant fungi, many of which are opportunistic human pathogens (Gostinčar et al. 2011). In the light of the small number of appropriate antifungal agents, and as both *R. mucilaginosa* and *C. parapsilosis* have been reported to be new emerging pathogens that can cause primarily catheter‐related infections and opportunistic nosocomial fungemia in immunocompromised patients (Neofytos et al. 2007; Pfaller et al. 2007; van Asbeck et al. 2009; Miceli et al. 2011), these data show promise for the development of new types of biodegradable detergents based on plant extracts.

## Conclusions

*Celtis australis* L. has to date been valued mainly for its wood. Hackberry fruit are thus small, and they have a relatively large stone, with the consequent low yield of mesocarp. These fruits are seldom consumed fresh, although sometimes they are used to produce liqueurs by mixing them with fruit brandy. Sensory properties and mineral composition of hackberry fruits are also similar to that of figs.

Our data show that these fruits are interesting from the nutritional point of view, as they contain dietary fiber, phenols, vitamins (tocopherols, carotenoids), and minerals. The amounts of total fiber and protein are comparable to those observed for figs.

The antioxidant activities of the water and ethanol extracts studied here increased with the amounts of total phenols that they contained. This study revealed the presence of epicatechin, gallic acid, vanillic acid, 3,5 DHA, delphinin‐3,5‐di‐*O*‐glucoside, cyanidin‐3,5‐di‐*O*‐glucoside, and pelargonidin‐3,5‐di‐*O*‐glucoside in these extracts. As the concentrations of these phenols varied according to the growth stage, the knowledge gained here can be applied to determine the optimal harvest time to maximize the yields of such bio‐active phenolics. Additionally, these fruits are a rich source of water‐soluble polyphenolic compounds, which are here identified, and some publications have indicated that leaves of *C. australis* contain the rare flavonoid c‐glycosides, such as acacetin 7‐O‐glucoside, isovitexin, and cytisoside.

Antimicrobial and, in particular, antifungal activities were also detected for the ethanol extracts prepared here from hackberry leaves, against the most important fungal opportunistic pathogen *C. albicans*, and against two emerging opportunistic pathogenic yeast *C. parapsilosis* and *R. mucilaginosa*. Further studies are thus needed to identify and define the active ingredients of these hackberry leaf ethanol extracts.

## Conflict of Interest

The authors declare that they have no conflicts of interest.
